# Papillary carcinoma on thyroglossal tract cyst: our attitude toward a rare pathology whose management is often controversial

**DOI:** 10.1093/jscr/rjad440

**Published:** 2023-08-08

**Authors:** Winga Foma, Solim U R Boko, Ingrid Douhadji, Essobiziou Amana, Essobozou P Pegbessou, Koffi Amegbor, Bathokedeou Amana, Essohanam Boko

**Affiliations:** ENT Department, Sylvanus Olympio University Hospital, Lomé, Togo; ENT Department, Sylvanus Olympio University Hospital, Lomé, Togo; ENT Department, Sylvanus Olympio University Hospital, Lomé, Togo; ENT Department, Sylvanus Olympio University Hospital, Lomé, Togo; ENT Department, Lomé-Commune Regional Hospital, Lomé, Togo; Anatomopathology Department, Sylvanus Olympio University Hospital, Lomé, Togo; ENT Department, Sylvanus Olympio University Hospital, Lomé, Togo; ENT Department, Campus University Hospital, Lomé, Togo

**Keywords:** thyroglossal tract cyst, papillary carcinoma, Sistrunk procedure, thyroidectomy

## Abstract

Malignant degeneration on remnants of the thyroglossal tract is a very rare phenomenon. In our practice setting, we report the management of papillary carcinoma on a thyroglossal tract cyst. This was a 44-year-old female patient with a postoperative diagnosis of papillary carcinoma of the thyroglossal tract with an atypical clinical and ultrasound presentation. She subsequently underwent total thyroidectomy and bilateral recurrent lymph node dissection, with resection of fibrous scar tissue in the previously operated hyoid region. We have discussed our therapeutic attitude to this rare pathology, the management of which is the subject of controversy.

## INTRODUCTION

The thyroglossal tract cyst (TTC) is a cervical mass resulting from persistent epithelial tissue of the thyroglossal duct that enlarges because of inflammation, infection and mucus retention [1]. It can occur anywhere along the midline between the foramen caecum and the thyroid gland. TTCs are most often found below the level of the hyoid bone (85%), as reported by Amana *et al*. [2] in Togo. Although more commonly found in the pediatric population, it also occurs in the adult population, with variable frequency. Malignant degeneration on remnants of the thyroglossal tract is a very rare phenomenon. It accounts for 1% of cysts operated on, with papillary carcinoma as the most common histological type [3]. We report on the management of papillary carcinoma on TTC in our context of a country with limited resources.

## CASE REPORT

**Figure 1:**
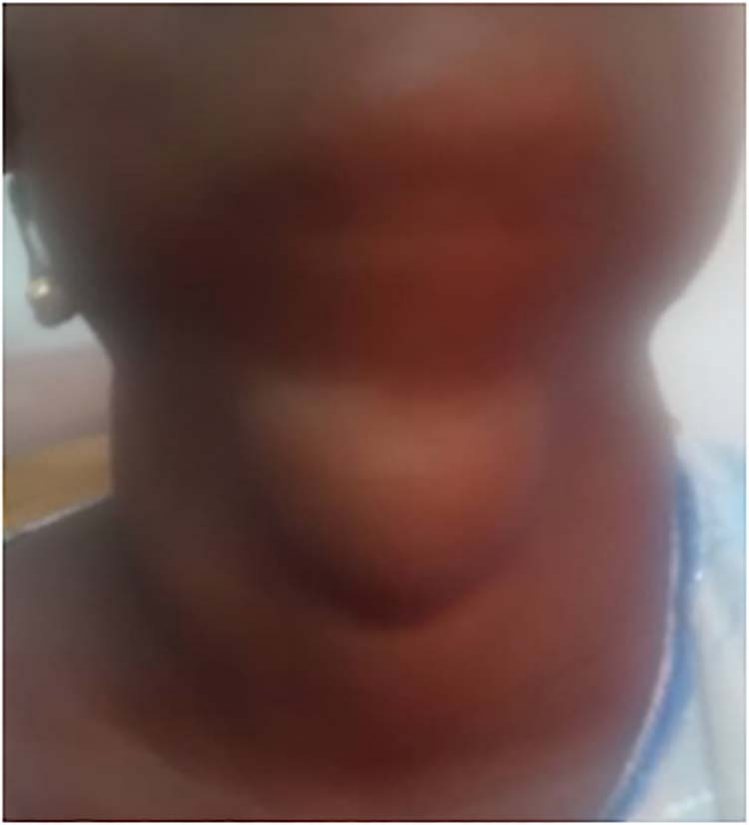
Median sub-hyoid swelling in our patient.

**Figure 2:**
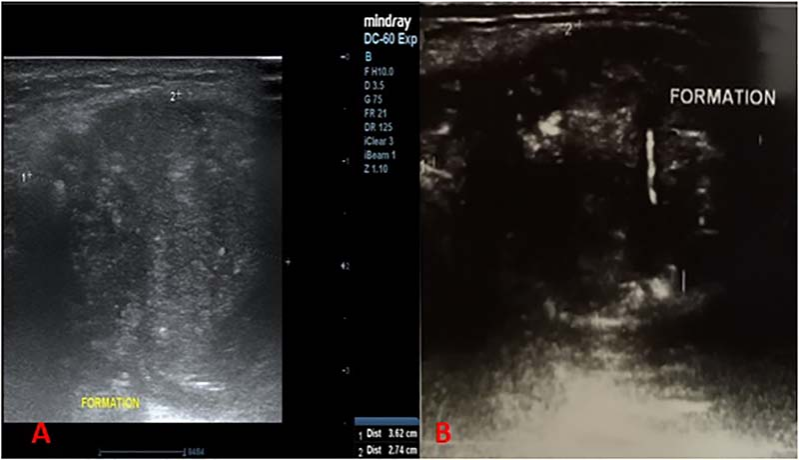
Longitudinal cervical ultrasound sections showing a heterogeneous formation, with more or less regular contours, associated with microcalcifications (**A**) with liquid portions showing posterior enhancement (**B**).

This was a 44-year-old female patient, with no specific pathological history, who consulted for a painless, median, anterior cervical swelling that had been progressively evolving for 2 and a half years. There was no evidence of dysphonia, dysphagia or cough. Physical examination revealed a median tumefaction, in contact with the sub-hyoid bone, oval in shape with a transverse major axis of about 6 cm, rising on swallowing and tongue protraction, painless, firm to palpation, regular in outline, with no inflammatory signs (Fig. 1). We suggested atypical TTC, dermoid cyst or ectopic thyroid nodule as diagnostic hypotheses, and requested further investigations. Cervical ultrasonography revealed a heterogeneous oval formation with internal calcifications, more or less regular contours, posterior reinforcement, relatively thick-walled, anterior cervical location, pushing back the muscles laterally and posteriorly, poorly vascularized on Doppler (Fig. 2A and B). The thyroid gland was normal in size, with a finely nodular echostructure.

In view of the close relationship with the hyoid bone, the frequency of TTCs in the anterior high cervical region and the presence of a cystic contingent within the lesion on ultrasound, the diagnosis of atypical TTC was retained.

**Figure 3:**
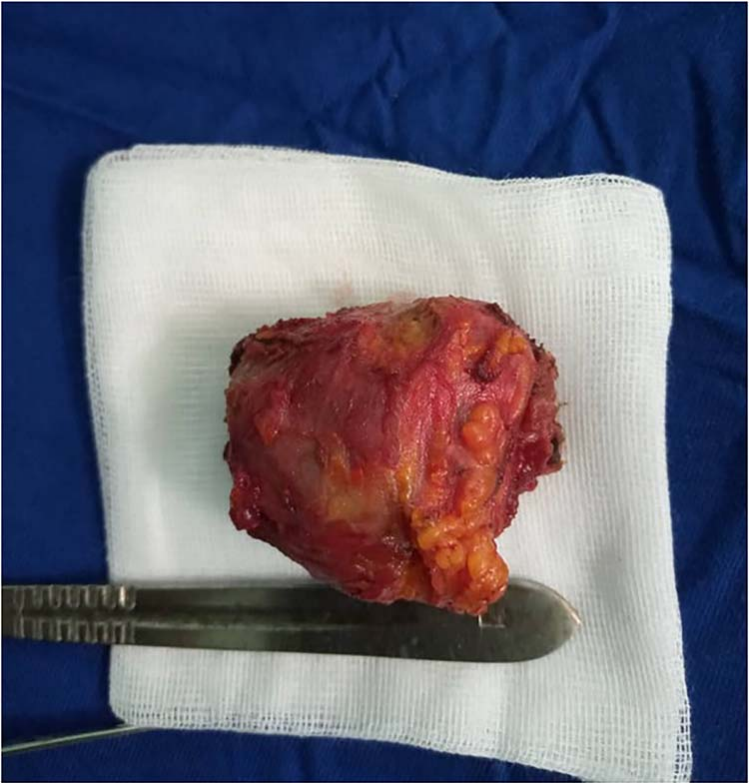
Surgical specimen after Sistrunk procedure.

**Figure 4:**
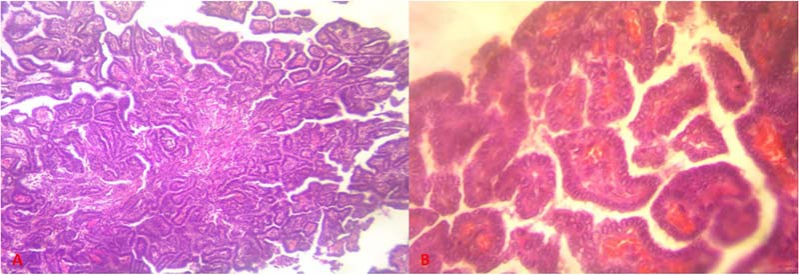
Histological sections showing papillary tumor proliferation ((**A**) = hematoxylin–eosin staining, magnification ×10), made up of irregularly branched small papillae, lined with cells with clear, vitreous cytoplasm, without true incised nuclei, with edematous, congestive conjunctival-vascular axes ((**B**) = hematoxylin–eosin staining, magnification ×40).

The patient was operated on using the Sistrunk procedure. Intraoperatively, the sub-hyoid muscles had adhered to the firm formation, with a thick wall connected to the hyoid bone by its upper pole and to the thyroid isthmus by a lower cord. The formation was removed as a monobloc, removing the body of the hyoid bone and adherent sub-hyoid muscles (Fig. 3). Postoperative management was straightforward. Anatomopathological examination of the specimen revealed a malignant tumor proliferation of papillary architecture, with no invasion of the cystic wall (Fig. 4A and B).

The postoperative diagnosis of papillary carcinoma on TTC was accepted. Six weeks later, the patient underwent total thyroidectomy and bilateral recurrent lymph node dissection, with resection of scar tissue in the hyoid region. The postoperative course was simple. Corrected serum calcium levels were normal, and the patient was treated with levothyroxine 125 ug/day, with a TSH target of 0.5 mIU/l. Anatomopathological examination of the thyroid gland, lymph node removal product and fibrous scar tissue revealed no carcinomatous lesions. Clinical, radiological and ultrasound follow-up was decided every 6 months. To date, after more than 3 years, no tumor recurrence has been noted.

## DISCUSSION

The first case described in our country, malignant degeneration of the TTC is rare. Its prevalence varies from 1 to 1.5% of cysts [4]. As in the present case, malignant TTC predominates in the female sex and the mean age of onset is in the fourth decade of life [5]. The clinical presentation is generally similar to that of a simple TTC, which explains why it is most often discovered by chance following an anatomopathological examination of the surgical specimen. However, a number of clinical signs should prompt the practitioner to suspect a possible neoplastic process, in particular the hard, fixed and/or irregular nature of the cervical mass, rapid increase in size or association with cervical lymph nodes [4]. On a paraclinical level, ultrasound findings that should raise suspicion of neoplasia within the TTC include solidity, the presence of intra-cystic calcifications and/or vegetations, and wall invasion [5]. Fine needle aspiration cytology may be indicated, except that it is only of value when it is positive, its positive predictive value being around 50% [6]; it could have helped us make the decision for one-stage surgery [5], but could not be performed in our patient.

Many authors believe that these carcinomas develop de novo within the TTC. The origin would be normal thyroid tissue present both in the cyst wall and along the entire path of the tract. Others suggest that the thyroglossal duct is a natural pathway for the spread of carcinomas from the thyroid gland [7, 8]. These theories underlie the disparities encountered in the therapeutic management of this type of pathology [9].

The Sistrunk procedure remains the gold standard for surgical excision of TTC, which is associated with a relatively low rate of cystic recurrence. The role of total thyroidectomy is controversial, and there is no consensus on the subject in cases of TTC malignancy. Some teams are satisfied with the total removal of the cyst using the Sistrunk procedure, and see no point in performing a total thyroidectomy, whereas others recommend completing the initial surgical procedure with a total thyroidectomy; still, others perform both procedures in a single operation if the preoperative diagnosis has been established by fine needle aspiration cytology, or if there is an associated thyroid nodule. The reasons given for the maximalist attitude are the frequency of association of degenerated TTCs with primary thyroid carcinomas, which varies from 11 to 40%, and the guarantee of better follow-up [2, 9]. Authors who find no benefit in thyroidectomy for the patient, cite as arguments the increased morbidity resulting from iterative interventions, the possibility of effective follow-up with re-intervention at a later stage in the event of thyroid cancer being discovered, and finally the good prognosis. These minimalist authors then reserve the completeness of the surgical procedure for patients considered to be at high evolutionary risk: age over 45, history of exposure to radiation, presence of nodule in the thyroid on radiological evaluation, presence of clinical or radiological lymph nodes, tumor over 1.5 cm in diameter at the level of the TTC, invasion of the cyst wall or positive margins on histopathological examination, lymph node metastases [5, 7]. In our case, despite the large size of the lesion and the finely nodular thyroid, the patient underwent a second stage of total thyroidectomy and bilateral recurrent lymph node dissection, mainly because of the context of our practice, where long-term follow-up is generally difficult, with patients often being lost to follow-up, and also because of the unavailability of the nuclear medicine technical platform in case scintigraphy or even radioactive iodine treatment was required. This therapeutic attitude to the difficulty of follow-up has already been suggested by other authors [10].

## CONCLUSION

Malignant degeneration of a TTC is rare and is often papillary carcinoma. Its clinical and paraclinical presentation is never specific. The diagnosis is most often made after an anatomopathological study of the surgical specimen. Management is comparable to that of differentiated thyroid cancer but remains controversial. The context of developing countries, with all the follow-up difficulties that medical staff is confronted with in relation to patients, justifies the broadness of the surgical gesture in the present case.
